# Optimization of Ultrasound-Assisted Extraction of Natural Antioxidants from the Flower of *Jatropha integerrima* by Response Surface Methodology

**DOI:** 10.3390/molecules21010018

**Published:** 2015-12-24

**Authors:** Dong-Ping Xu, Yue Zhou, Jie Zheng, Sha Li, An-Na Li, Hua-Bin Li

**Affiliations:** 1 Guangdong Provincial Key Laboratory of Food, Nutrition and Health, School of Public Health, Sun Yat-Sen University, Guangzhou 510080, China; xudongping1989@163.com (D.-P.X.); zhouyue3@mail2.sysu.edu.cn (Y.Z.); zhengj37@mail2.sysu.edu.cn (J.Z.); lianna28@163.com (A.-N.L.); 2 School of Chinese Medicine, The University of Hong Kong, Hong Kong, China; lishasl0308@163.com

**Keywords:** *Jatropha integerrima*, flower, natural antioxidant, ultrasound-assisted extraction, response surface methodology

## Abstract

An ultrasound-assisted extraction (UAE) method was developed for the efficient extraction of natural antioxidants from the flowers of *Jatropha integerrima*. Four independent variables, including ethanol concentration, solvent/material ratio, ultrasound irradiation time and temperature were studied by single factor experiments. Then, the central composite rotatable design and response surface methodology were employed to investigate the effect of three key parameters (ethanol concentration, solvent/material ratio, and ultrasound irradiation time) on the antioxidant activities of the flower extracts. The optimal extraction conditions were an ethanol concentration of 59.6%, solvent/material ratio of 50:1, ultrasound irradiation time of 7 min, and ultrasound irradiation temperature of 40 °C. Under these conditions, the optimized experimental value was 1103.38 ± 16.11 µmol Trolox/g dry weight (DW), which was in accordance with the predicted value (1105.49 µmol Trolox/g DW). Furthermore, the antioxidant activities of flower extracts obtained by UAE were compared with those produced by the traditional maceration and Soxhlet extraction methods, and UAE resulted in higher antioxidant activities after a shorter time at a lower temperature. The results obtained are helpful for the full utilization of *Jatropha integerrima*, and also indicate that ultrasound-assisted extraction is an efficient method for the extraction of natural antioxidants from plant materials.

## 1. Introduction

Antioxidants play an important role in preventing or slowing down autoxidation in biological systems and food, and have attracted much attention [1]. Naturally-occurring antioxidants in plants like flavonoids and phenolic acids are an important part of the human diet, and are of considerable interest due to their capacity for scavenging free radicals like superoxide anion radical and hydroxyl radical, which can cause several diseases, such as cancer, arteriosclerosis, autoimmune and neurodegenerative syndromes [2,3,4,5,6]. Besides, natural antioxidants from herbs and spices could preserve the quality of food products from chemical oxidation and lipid rancidity as more safety and healthy additives compared with synthetic ones [1,6,7]. Thus, evaluation, extraction, separation and purification of natural antioxidants are very important. Furthermore, effective extraction of antioxidants from plant materials is very helpful for full utilization of natural resources.

*Jatropha integerrima*, a member of the Euphorbiaceae family, is a drought tolerant perennial shrub. It flowers throughout the year, and is one of the most important ornamental species cultivated in the tropics and subtropics for its attractive crimson flowers [8,9]. The essential oils of the leaves and seeds of *Jatropha integerrima* displayed weak antimicrobial activity against *Bacillus cereus* and *Staphylococcus aureus* [10]. In another study, two cyclic peptides isolated from latex of *Jatropha integerrima* exhibited significant cytotoxic activity against KB human nasopharyngeal carcinoma cells *in vitro* [11]. Besides, diterpenoids isolated from the trunks of *Jatropha integerrima* exhibited stronger inhibitory activity against thioredoxin reductase than the positive control (curcumin), which is a potential target for cancer chemotherapy with redox balance and antioxidant functions [12]. Lately, it was found that flower of *Jatropha integerrima* had the strongest antioxidant capacity among 51 edible and wild flowers from China [13], which implied that the flowers of *Jatropha integerrima* were a potential rich resource of natural antioxidants. Consequently, effective extraction of antioxidants from flowers *of Jatropha integerrima* should be helpful for its full utilization.

The conventional methods of antioxidant extraction from plant materials are mainly maceration and Soxhlet extraction, which are very time-consuming and require relatively large quantities of toxic organic solvents. Ultrasound-assisted extraction (UAE) has been found to be a more effective and environmentally friendly way of extracting natural antioxidants from plant materials for its characteristics of shorter extraction time and less use of organic solvents [14,15,16,17,18,19,20]. The acoustic cavitation effect of UAE permits better penetration of the solvent into the sample, increasing the extraction yield of target components. Because the efficiency of an extraction process is usually influenced by several factors, such as solvent concentration, solvent to solid ratio, extraction temperature and time, optimization of these parameters is very important to obtain high extraction yields.

Response surface methodology (RSM) is an efficaceous mathematical and statistical technique for simultaneously evaluating the interaction of several experimental parameters [6,20]. In the present study, in order to maximize the extraction of antioxidants from the flowers of *Jatropha integerrima* by UAE, the effects of several extraction parameters (ethanol concentration, solvent/material ratio, ultrasound irradiation time and temperature) were investigated and the optimal conditions were obtained using response surface methodology (RSM) by employing a five-level, three-variable central composite rotatable design (CCRD). Additionally, to further confirm the extraction efficiencies, a comparison between UAE and two conventional extraction methods (maceration and Soxhlet extraction) was also conducted.

## 2. Results and Discussion

### 2.1. Single Factor Experiment

In the preliminary study, the influence of several factors (ethanol concentration, solvent/material ratio, ultrasonic time and temperature) on antioxidant activities of the extract of *Jatropha integerrima* flower was detected and analyzed.

#### 2.1.1. Effect of Ethanol Concentration

**Figure 1:**
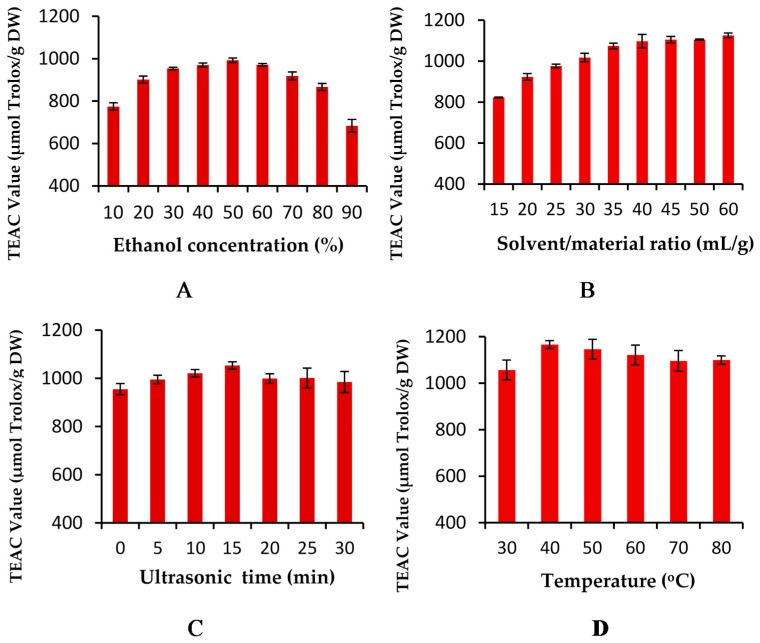
Effects of extraction parameters on the antioxidant activities of flower extracts: (**A**) Effect of ethanol concentration on the antioxidant activities; (**B**) Effect of the solvent/material ratio on the antioxidant activities; (**C**) Effect of ultrasound irradiation time on the antioxidant activities; and (**D**) Effect of temperature on the antioxidant activities.

Methanol, ethanol, acetone and isopropanol, with different levels of water, have been widely used to extract antioxidant components from botanical materials, especially herbs [21,22,23,24,25]. Compared with methanol, acetone and isopropanol, ethanol and water are safer to human beings and the environment. Thus, aqueous ethanol was used in this study, and the results are shown in Figure 1A. Other extraction conditions were set as follows: solvent/material, 100 mL/g; ultrasound irradiation time, 30 min; temperature, 30 °C. When the ethanol concentration increased from 10% to 50% (*v*/*v*), the total antioxidant activities of the extracts increased significantly, changing from 774.40 ± 17.44 to 992.36 ± 10.48 µmol Trolox/g DW. An obvious decrease was observed with further increases of the ethanol concentration from 50% to 90%. The total antioxidant activities using 60% and 90% ethanol were 971.05 ± 6.05 µmol Trolox/g DW and 683.34 ± 23.83 µmol Trolox/g DW, respectively. A similar result was reported in the extraction of antioxidant compounds from *Zizyphus lotus* fruits [6]. The results indicate that 50% ethanol was more suitable in the subsequent experiments.

#### 2.1.2. Effect of Solvent/Material Ratio

To study the effect of different liquid–solid ratios on the antioxidant activities of the extract of *Jatropha integerrima* flowers, different solvent/material ratios (15:1, 20:1, 25:1, 30:1, 35:1, 40:1, 45:1, 50:1, 60:1, mL/g) were employed, and the other extraction conditions were set as follows: ethanol concentration, 50%; ultrasound irradiation time 30 min; ultrasound irradiation temperature, 30 °C. The results are displayed in Figure 1B. When the ratio of solvent to material increased from 15:1 to 40:1, the total antioxidant activities increased by 33% (from 742.46 ± 2.22 to 990.17 ± 32.96 µmol Trolox/g DW). When the solvent/material ratio exceeded 40:1 (mL/g), the total antioxidant activities almost did not change. The reason was that the higher ratio of solvent to material could accelerate mass transfer and facilitate the diffusion of antioxidants into the medium until the mass transfer process reached its maximum [26,27]. Therefore, 40:1 was selected as optimal solvent/material ratio.

#### 2.1.3. Effect of Ultrasound Irradiation Time

The effect of different ultrasound irradiation times on the total antioxidant activities of the extracts was compared, and the results are shown in Figure 1C. Other extraction conditions were set as follows: ethanol concentration, 50%; ratio of solvent to material, 40 mL/g; ultrasound irradiation temperature, 30 °C. The antioxidant activities increased from 0 to 15 min, and then decreased when the ultrasound irradiation time was longer than 15 min. The maximum antioxidant activities (1053.20 ± 15.66 µmol Trolox/g DW) were obtained after 15 min. The results indicate that under the ultrasound irradiation treatment, the diffusion of the bioactive compounds from material to solvent might be improved and the equilibrium for dissolution might be established in a short time. But the antioxidant components might be degraded after a long exposure to ultrasonic irradiation [28,29]. Thus, 15 min was used in the subsequent experiments.

#### 2.1.4. Effect of Ultrasound Irradiation Temperature

The effect of temperature on antioxidant activities of the extracts was evaluated, and the results are shown in Figure 1D. Other extraction conditions were set as follows: ethanol concentration, 50%; solvent/material ratio, 40 mL/g; ultrasound irradiation time, 15 min. The antioxidant activities was improved when the temperature was raised from 30 to 40 °C, then the antioxidant activities decreased from 40 to 80 °C. The maximum antioxidant activity (1166.22 ± 16.89 µmol Trolox/g) could be obtained at 40 °C. The results indicate that natural antioxidants from *Jatropha integerrima* reached an equilibrium of desorption and solubility at 40 °C, and some thermally unstable antioxidants from the flowers could be decomposed at higher temperature [21,30]. Therefore, 40 °C was used in the subsequent experiments.

### 2.2. Response Surface Methodology

In order to evaluate interaction of several experimental parameters, response surface methodology is used.

#### 2.2.1. Experimental Design and Results of CCRD

According to the single factor experiment results, an ethanol concentration of 50% (*v*/*v*), solvent/material ratio of 40:1 (mL/g), and ultrasound irrdiation time of 15 min were chosen as the central condition of the central composite experiment design, and the effects of three independent variables on the dependent variable (TEAC value) at five levels were investigated. The 20 experimental designs and the results are shown in Table 1. Results show that the antioxidant activities ranged from 900.78 to 1092.03 μmol Trolox/g DW. The maximum antioxidant activity was recorded under the experimental parameters of ethanol concentration of 50%, solvent/material ratio of 56.8:1 and ultrasound irradiation time of 15 min.

**Table 1:** Experimental design of response surface analysis and its experimental values.

Run	X_1_ (Concentration of Ethanol, %)	X_2_ (Solvent/Material Ratio, mL/g)	X_3_ (Extraction Time, min)	Y (TEAC Value, µmol Trolox/g DW)
1	70	50	7	1082.39
2	83.64	40	15	952.35
3	50	40	15	1063.53
4	50	40	15	1086.78
5	30	30	23	977.83
6	50	23.18	15	951.72
7	50	40	15	1051.91
8	70	30	7	960.39
9	50	56.82	15	1092.03
10	30	50	23	1000.05
11	50	40	28.45	1009.28
12	50	40	15	1040.28
13	50	40	15	1071.28
14	70	50	23	1029.11
15	50	40	15	1055.78
16	70	30	23	948.77
17	30	30	7	919.71
18	50	40	1.55	1063.53
19	16.36	40	15	900.78
20	30	50	7	990.36

#### 2.2.2. Fitting the Model

Analysis of variance (ANOVA) was performed to evaluate the quality of the fitted model (Table 2). In this model, the second-order polynomial model for the extraction of antioxidants was statistically significant with a small model *p*-value (*p* < 0.0001) and satisfactory coefficient of determination (R^2^ = 0.965). The linear parameters (X_1_, X_2_) and quadratic parameters (X_1_^2^, X_2_^2^) were significant at the level of *p* < 0.01, interaction parameters (X_1_X_2_, X_1_X_3_) and quadratic parameter (X_3_^2^) were significant at the level of *p* < 0.05. The “Lack of Fit-Value” of the model is not significant with a *p*-value of 0.61. The significant regression and non-significant lack of fit indicated that the regression equation is adequate to represent the actual relationship between the response values (Y) and three independent variables. The quadratic regression equation was obtained as follows Equation (1):

Y = 285.63 + 11.68X_1_ + 14.21X_2_ + 14.41X_3_ + 0.07X_1_X_2_ − 0.10X_1_X_3_ − 0.14X_2_X_3_ − 0.12X_1_^2^ − 0.15X_2_^2^ − 0.15X_3_^2^ (1)

**Table 2:** ANOVA for the response surface quadratic model.

Source	Sum of Squares	df	Mean Square	*F* Value	*p* Value	Significant
Model	64580.50	9	7175.61	30.93	<0.0001	significant
X_1_ (ethanol concentration)	3526.02	1	3526.02	15.20	0.0030	
X_2_ (ratio of solvent to material)	20659.29	1	20659.29	89.04	<0.0001	
X_3_ (ultrasonic time)	571.30	1	571.30	2.46	0.1477	
X_1_X_2_	1497.85	1	1497.85	6.46	0.0293	
X_1_X_3_	2201.69	1	2201.69	9.49	0.0116	
X_2_X_3_	1014.57	1	1014.57	4.37	0.063	
X_1_^2^	33523.51	1	33523.51	144.48	<0.0001	
X_2_^2^	3044.43	1	3044.43	13.12	0.0047	
X_3_^2^	1272.59	1	1272.59	5.48	0.0412	
Residual	2320.28	10	232.03			
Lack of Fit	1006.46	5	201.29	0.77	0.6115	Not significant
Pure Error	1313.82	5	262.76			
Cor Total	66900.79	19				
R-Squared	0.965					
Adj R-Squared	0.934					

#### 2.2.3. Analysis of Response Surfaces

**Figure 2:**
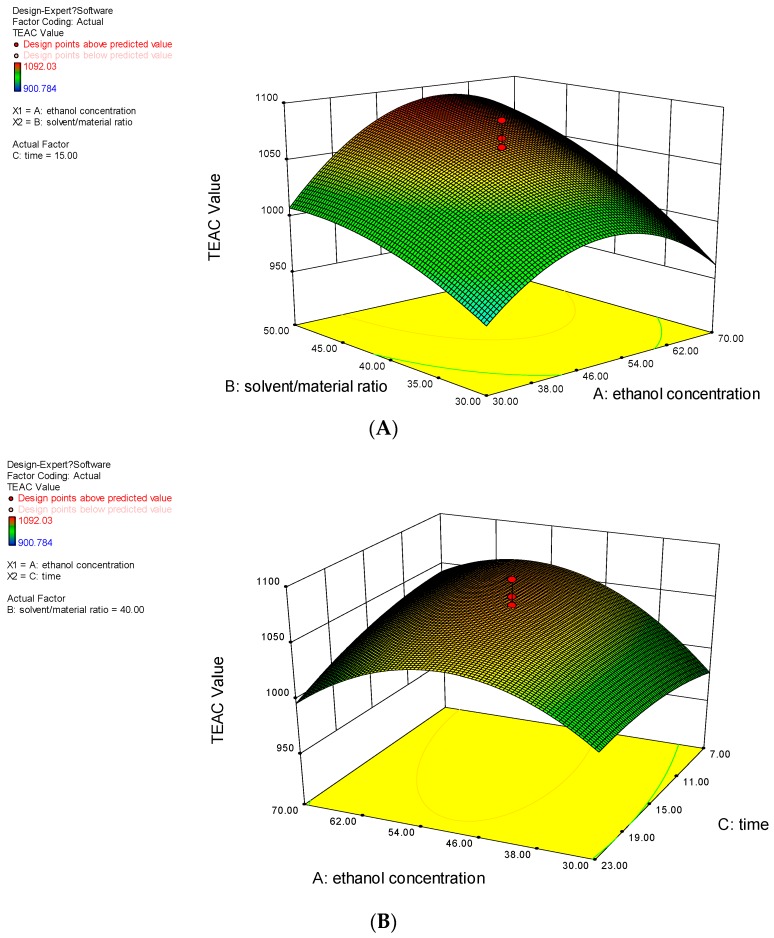

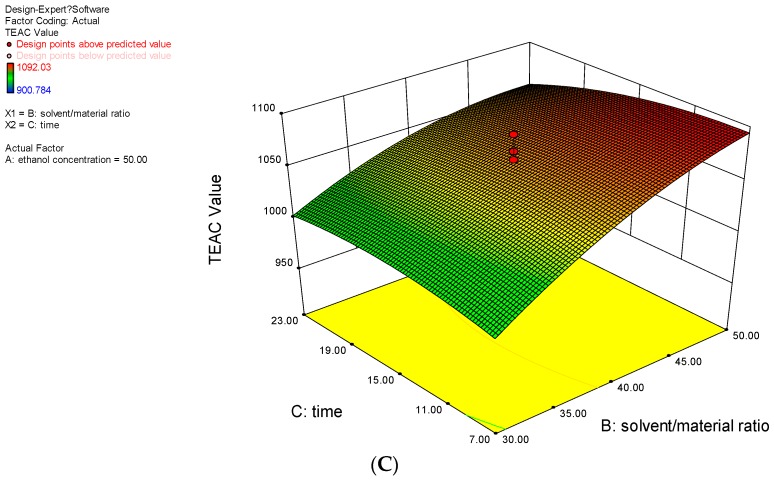
Interaction effects of ethanol concentration and solvent/material ratio (**A**); ethanol concentration and ultrasound irradiation (**B**); solvent/material ratio and ultrasound irradiation time (**C**) on antioxidant activities.

Response surface plots are shown in Figure 2. The interaction between various factors can be seen directly from the response surfaces plots. Figure 2A shows the effect of the interaction of ethanol concentration and solvent/material ratio on the antioxidant activities at a fixed ultrasound irradiation time of 15 min. An increase of liquid-to-solid ratio (X_2_) resulted in an increase of antioxidant activities to a maximum at a certain level, while an increase of ethanol concentration (X_1_) resulted in an initial increase of antioxidant activities and then decreased as the concentration continued to increase. Figure 2B shows the effect of the interaction of ethanol concentration and ultrasound irradiation time on the antioxidant activities at a fixed solvent/material ratio of 40:1 mL/g. It could be observed that the concentration of ethanol resulted in similar effects on the antioxidant activities as in Figure 2A, whereas ultrasound irradiation time had only a limited impact on the antioxidant activities. Figure 2C shows the effect of the interaction of solvent/material ratio and ultrasound irradiation time on the antioxidant activities at a fixed ethanol concentration of 50%. It could be observed that the solvent/material ratio demonstrated a strongly positive influence on the antioxidant activities, whereas ultrasound irradiation time had only a slight impact. The combination of the analysis of variance (ANOVA) (Table 2) and response surfaces (Figure 2) indicated that the interaction effect between ethanol concentration and solvent/material ratio (X_1_X_2_), and ethanol concentration and ultrasound irradiation time (X_1_X_3_) were statistically significant, but the interaction effect between solvent/material ratio and ultrasound irradiation time (X_2_X_3_) was non-significant. Furthermore, it was concluded that the effect of ethanol concentration and solvent/material ratio were more significant than ultrasound irradiation time on the antioxidant activities.

#### 2.2.4. Verification of Predicted Value of the Models

The optimal conditions obtained using the model were as follows: ethanol concentration, 59.6%; solvent/material ratio, 50:1; temperature, 40 °C; and ultrasound irradiation time, 7 minutes. Under optimal conditions, the maximum response value of 1105.49 µmol Trolox/g DW was predicted by the model. Verification experiments were performed at the predicted conditions. The result showed that the experimental value (1103.376 ± 16.11 µmol Trolox/g DW; *n* = 6) was consistent with the predictive value (Table 3). The good correlation between predicted and experimental value demonstrated that response surface methodology is accurate and reliable to find the optimum ultrasound irradiation extraction conditions for antioxidants from flowers of *Jatropha integerrima*.

**Table 3:** Optimum conditions, predicted and experimental value.

Optimal Condition			TEAC Value (µmol Trolox/g DW)	
Ethanol Concentration (%)	Solvent/Material Ratio (mL/g)	Time (min)	Experimental	Predicted
59.6	50	7	1103.38 ± 16.11	1105.49

### 2.3. Comparison of Ultrasound-Assisted Extraction with Maceration and Soxhlet Extraction Methods

As shown in Table 4, the antioxidant activity by UAE (1103.38 ± 16.11 µmol Trolox/g) was 1.88 times that obtained by Soxhlet extraction (588.06 ± 10.47 µmol Trolox/g), and the extraction temperature of UAE (40 °C) was also greatly reduced compared to Soxhlet extraction (95 °C). Besides, the ultrasound irradiation time of UAE (7 min) was significantly shorter than maceration method (24 h) and Soxhlet extraction (4 h). It was concluded that UAE was the most efficient extraction method among the three methods. The results were in agreement with published studies on the extraction of nutraceutical compounds from *Dendrobium candidum*, flavonoids from grapefruit (*Citrus paradisi* L.) solid wastes and anthocyanins from blackberry and sweet cherry cultivars [31,32,33], definitely demonstrating that UAE can be applied in the extraction of antioxidants from *Jatropha integerrima* with higher efficiency and shorter time.

**Table 4:** The comparison of UAE with maceration and Soxhlet extraction.

Extracting Method	Ethanol Concerntration	Temperature	Time	TEAC Value (µmol Trolox/g DW)
UAE	59.63%	40 °C	7 min	1103.38 ± 16.11
Maceration extraction	59.63%	25 °C	24 h	1022.65 ± 42.32
Soxhlet extraction	59.63%	95 °C	4 h	588.06 ± 10.47

## 3. Experimental Section

### 3.1. Chemicals

The compounds 6-hydroxy-2,5,7,8-tetramethylchromane-2-carboxylic acid (Trolox), and 2,2′-azinobis(3-ethylbenothiazoline-6-sulphonic acid) diammonium salt (ABTS) were purchased from Sigma-Aldrich (St. Louis, MO, USA). Potassium persulfate was purchased from Tianjin Chemical Factory (Tianjin, China). Ethanol and methanol were obtained from Kelong Chemical Factory (Chengdu, China). All chemicals used in the experiments were of analytical grade, and deionized water was used.

### 3.2. Instruments

The ultrasound-assisted extraction was carried out in a Kj1012B ultrasonic water bath (Guangzhou Kejin Ultrasonic Instrument Factory, Guangzhou, China) with an electric power of 400 W, and 40 kHz frequency, equipped with a digital timer and a temperature controller for the control of time and temperature. The bath consisted of a rectangular vessel (20.2 cm × 17.5 cm × 22.9 cm). The ultrasonic energy was delivered from the bottom to the water with a relatively constant frequency of 40 kHz.

### 3.3. Sample Preparation

The flowers of *Jatropha integerrima* were picked from Guangzhou, China. The flowers were washed with deionized water, given an airing at room temperature, and dried to 2.1% residual moisture utilizing freeze-drying technology. Then, the flowers were ground into fine particles (smaller than 0.3 mm) using a special grinder for processing food (RHP-100, Ronghao Industry & Trade Co. Ltd., Yongkang, China), and were stored at 4 °C until used.

### 3.4. Extraction Procedures of Antioxidants

#### 3.4.1. Ultrasound-assisted Extraction

The ground powder (0.100 g) was placed in a capped tube (15 mL) and mixed with an appropriate amount of ethanol-water solution (according to the experimental design). Next, the mixture was immersed in water in the ultrasonic device at 40 kHz and fixed well in the same position during sonication, for the pre-set ultrasound irradiation time and temperature with a power of 28 W/L. The real temperature of the extraction solution was monitored by a thermometer, and the target temperature was adjusted/controlled using the temperature controller. After extraction, the sample was centrifuged at 4200 *g* for 30 min, and the supernatant was obtained and stored at 4 °C for the antioxidant activity determination within 48 h.

#### 3.4.2. Maceration Extraction

The ground powder (0.100 g) was mixed with 59.63% ethanol (5 mL), and the antioxidants were extracted at 25 °C for 24 h in a shaking water bath. Then, the sample was centrifuged at 4200 *g* for 30 min, and the supernatant was collected and stored at 4 °C for the antioxidant activity determination within 48 h.

#### 3.4.3. Soxhlet Extraction

The ground powder (2.000 g) were kept over a Whatman filter paper. The antioxidants were extracted with 59.63% ethanol (400 mL) during percolation. The solvent was heated at 95 °C in a Soxhlet extractor [27,34]. After 4 h, the extract was taken out and stored at 4 °C for the antioxidant activity determination within 48 h.

### 3.5. Determination of Antioxidant Capacity 

The antioxidant activity of the extract was evaluated using Trolox equivalent antioxidant capacity assay according to the procedure described previously with slight modification [35]. Firstly, the ABTS^•+^ stock solution was prepared through mixing 7 mmol/L ABTS with 2.45 mmol/L potassium persulfate in a volume ratio of 1:1, and incubating in the dark for 16 h at room temperature and used within 2 days. Secondly, the ABTS^•+^ stock solution were dilute to make sure that the absorbance of ABTS^•+^ working solution was 0.70 ± 0.05 at 734 nm. Finally, 100 μL of the dilute sample was mixed with 3.8 mL ABTS^•+^ working solution and the absorbance was measured at 734 nm after 6 min of incubation at room temperature. The results were calculated and expressed as µmol Trolox/g dry weight.

### 3.6. Experimental Design

#### 3.6.1. Single-Factor Experiments

To evaluate the effect of each factor under ultrasound treatment on antioxidant activity of the extract of *Jatropha integerrima* flower, ethanol concentration (0%, 10%, 20%, 30%, 40%, 50%, 60%, 70%, 80%, 90%), solvent/material ratio (15:1, 20:1, 25:1, 30:1, 35:1, 40:1, 45:1, 50:1, 60:1 mL/g), ultrasound irradiation time (0, 5, 10, 15, 20, 25, 30 min) and ultrasound irradiation temperature (30, 40, 50, 60, 70, 80 °C) were investigated as single factor variables in the experimental design. Three variables that affected the extraction efficiencies significantly were selected in this part for the subsequent experiments.

#### 3.6.2. Response Surface Methodology Experiments

Response surface method was used to find the optimal condition of ultrasound-assisted extraction. According to the results of single factor optimization, three variables were selected. Each variable was coded as X_1_–X_3_ and examined in five levels (Table 5). The 20 experimental runs including six replicates at the center point were employed. The Design-Expert (DE) design assumes that the main effects of the variables have interactions and are based on a second-order polynomial model [36,37], as follows:

Y = β_0_ ＋ ∑β_i_X_i_ ＋ ∑β_ii_ Xi^2^ ＋ ∑β_ij_ X_i_ X_j_ (2)

where Y is the response value; β_0_ is the constant; β_i_ is the linear regression coefficient; β_ii_ is the quadratic regression coefficient; β_ij_ is the interaction regression coefficient; X_i_ and X_j_ are the independent variables.

### 3.7. Statistical Analysis

All the experiments were performed in triplicate, and the average value ± SD (standard deviation) was reported. Statistical analysis was performed using Design Expert 8.06.1, and Excel 2007. 

**Table 5:** Variables and their five levels employed in central composite rotatable design.

Variable	Units	Symbol	Code Levels				
			−1.68	−1	0	1	1.68
Ethanol concentration	% (*v*/*v*)	X_1_	16.36	30	50	70	83.64
Solvent/material ratio	mL/g	X_2_	23.18	30	40	50	56.82
Ultrasonic time	min	X_3_	1.55	7	15	23	28.45

## 4. Conclusions

In this study, an ultrasound-assisted extraction method was developed for the extraction of natural antioxidants from the flower of *Jatropha integerrima*, and the optimal extraction conditions were obtained by response surface methodology. The high correlation (R^2^ = 0.965) of the model showed that the second-order polynomial model could successfully express the influence of independent variables on the response. The results showed that the extraction conditions including ethanol concentration, solvent/material ratio and ultrasound irradiation time markedly influenced the antioxidant activities of the extract of *Jatropha integerrima* flower. The optimal UAE conditions were as follows: ethanol concentration of 59.6%, solvent/material ratio of 50:1, ultrasound irradiation time of 7 min, and temperature of 40 °C, which resulted in 1103.38 ± 16.11 µmol Trolox/g DW for antioxidant activities of the extract of *Jatropha integerrima* flower. The comparison of UAE and two conventional extraction methods (maceration and Soxhlet extraction) showed the superiority of UAE for extracting antioxidants from the flower of *Jatropha integerrima*. It has been evidenced that UAE combined with RSM is an effective technique for the extraction of natural antioxidants from the flower of *Jatropha integerrima*. The results should be helpful for full utilization of *Jatropha integerrima*, and also indicate that UAE is an efficient method for the extraction of natural antioxidants from plant materials.
